# Deletion of a cyclin-dependent protein kinase inhibitor, *CsSMR1*, leads to dwarf and determinate growth in cucumber (*Cucumis sativus* L.)

**DOI:** 10.1007/s00122-021-04006-7

**Published:** 2021-11-29

**Authors:** Shuai Li, Qiqi Zhang, Huimin Zhang, Jie Wang, Jinjing Sun, Xueyong Yang, Sanwen Huang, Zhonghua Zhang

**Affiliations:** 1 Key Laboratory of Biology and Genetic Improvement of Horticultural Crops of the Ministry of Agriculture, Sino-Dutch Joint Laboratory of Horticultural Genomics, Institute of Vegetables and Flowers, Chinese Academy of Agricultural Sciences, Beijing, 100081 China; 2 Genome Analysis Laboratory of the Ministry of Agriculture, Agricultural Genomics Institute at Shenzhen, Chinese Academy of Agricultural Sciences, Shenzhen, 518124 China; 3 College of Horticulture, Qingdao Agricultural University, Qingdao, 266109 China

## Abstract

**Key message:**

A 7.9 kb deletion which contains a cyclin-dependent protein kinase inhibitor leads to determinate growth and dwarf phenotype in cucumber.

**Abstract:**

Plant architecture is a composite character which are mainly defined by shoot branching, internode elongation and shoot determinacy. Ideal architecture tends to increase the yield of plants, just like the case of “Green Revolution” increased by the application of semi-dwarf cereal crop varieties in 1960s. Cucumber (*Cucumis sativus* L.) is an important vegetable cultivated worldwide, and suitable architecture varieties were selected for different production systems. In this study, we obtained a novel dwarf mutant with strikingly shortened plant height and determinate growth habit. By bulked segregant analysis and map-based cloning, we delimited the *dw2* locus to a 56.4 kb region which contain five genes. Among all the variations between WT and *dw2* within the 56.4 kb region, a 7.9 kb deletion which resulted in complete deletion of *CsaV3_5G035790* in *dw2* was co-segregated with the dwarf phenotype. Haplotype analysis and gene expression analysis suggest that *CsaV3_5G035790* encoding a cyclin-dependent protein kinase inhibitor (*CsSMR1*) be the candidate gene responsible for the dwarf phenotype in *dw2*. RNA-seq analysis shows that several kinesin-like proteins, cyclins and reported organ size regulators are expressed differentially between WT and *dw2*, which may account for the reduced organ size in dwarf plants. Additionally, the down-regulation of *CsSTM* and *CsWOX9* in *dw2* resulted in premature termination of shoot apical meristem development, which eventually reduces the internode number and plant height. Identification and characterization of the *CsSMR1* provide a new insight into cucumber architecture modification to be applied to mechanized production system.

**Supplementary Information:**

The online version contains supplementary material available at 10.1007/s00122-021-04006-7.

## Introduction

Higher plants exhibit diverse architectures which are defined by the combination of shoot branching, internode elongation and shoot determinacy (Wang and Li 2008). Despite of external factors such as light and temperature, plant architecture is determined mainly by the genetic regulation factors. Crops with ideal architecture often have increased planting density, higher photosynthetic efficiency and better lodging-resistant ability, thus contributed to higher yield ultimately. Therefore, identifying of the architecture defective mutants and elucidation of its regulation mechanism will not only address the fundamental issues in plant science, but also facilitate the breeding of high-yield crops.

There are many factors affecting internode elongation, while most of them involve in the plant hormone biosynthesis or signal transduction pathway. Based on extensive studies with mutants displayed dwarf phenotype, two plant hormones, gibberellin (GA) and brassinosteroid (BR), are regarded as major factors that determine plant height. GAs is a large family of tetracyclic diterpenoid plant hormones that play important roles in multiple plant growth and developmental processes, especially in stem elongation. The semi-dwarf rice results from a deficiency in the *GA 20-oxidase* gene (*OsGA20ox2*) of the GA biosynthetic pathway, which brought the “Green Revolution” and increased rice yield significantly (Ashikari et al. 2002). *GIBBERELLIC ACID INSENSITIVE* (*GAI*) is a negative GA-response regulator in *Arabidopsis*, and its orthologs in wheat (*Rht*) and maize (*D8*) are the “Green Revolution” genes that have greatly enhanced grain yields since the 1960s and 1970s (Peng et al. 1999). In cucumber, *Csdw* mutant exhibited a dwarf phenotype with a reduced internode length that could be partially rescued through GA_3_ application, and endogenous GA_3_ levels from the stem of *Csdw* decreased distinctly (Xu et al. 2018). Although BRs was discovered relatively late, it plays an important role in plant architecture regulation. Plenty of mutants involved in BR biosynthesis and signaling process were identified in *Arabidopsis*, rice, maize and cotton (Clouse et al. 1996; Mori et al. 2002; Ren et al. 2020; Tanabe et al. 2005; Tian et al. 2019; Yang et al. 2014). In cucumber, *super compact-1* (*scp-1*), *super compact-2* (*scp-2*) and *compact plant architecture* (*cpa*) are BRs-deficient mutants which were identified to encode CYP85A1, CsDET2 and CsDWF5 respectively, (Hou et al. 2017; Wang et al. 2017; Zhang et al. 2021).

Shoot development in flowering plants is a continuous process ultimately controlled by the activity of the SAM (Sussex 1989). There are two types of shoot apical architecture in flowering plants: indeterminate and determinate. The main shoot apical of indeterminate plants grows indefinitely and produces lateral organs on its flanks, while the SAM of main axis completely converts into flowers in determinate plants. Shoot determinacy is an important plant architecture trait in cucumber. Cucumber varieties with determinate growth habit were preferable under open fields in North America for its labor-saving and once-over harvesting character, while the indeterminate growth habit varieties were more favorable under protected environment in East Asia and Europe for the fruits can be harvested continuously for an extended growth period. *TERMINAL FLOWER 1* (*TFL1*), *CENTRORADIALIS* (*CEN*) and *FLOWERING LOCUS T* (*FT*) belongs to the phosphatidyl ethanolamine-binding proteins (PEBPs) family and are key integrators of the floral transition. FT interacts with bZIP family transcription factor FD to promote floral development through transcriptional activation of *LFY* and *AP1* (Abe et al. 2005; Wigge Philip et al. 2005). *TFL1* is specifically expressed in the central region of apical meristem, but its protein spreads throughout the meristem and inhibits the expression of *LFY* and *AP1* to prevent plants from flowering (Conti and Bradley 2007). To date, *CsTFL1* and *CsCEN* have been elucidated to regulate growth habit in cucumber via similar regulatory mechanism (Njogu et al. 2020; Wen et al. 2019, 2021).

Coordination between cell cycle and cell differentiation is essential for proper development of multicellular organisms. The cell cycle transition is controlled by a conserved class of Ser/Thr kinases known as cyclin-dependent kinases (CDKs) between plants and animals (Veylder et al. 2003). CDK inhibitors (CKIs) negatively control cell cycle progression to prevent premature passage through checkpoints. There were two types of plant-specific CKI in land plants, known as Inhibitor/Interactor of CDC2 Kinase/KIP-related proteins (ICK/KRPs) and SIAMESE-related proteins (SMRs), of which SIM is the founding member. Among them, ICK/KRP members were reported to play diversity roles in root initiation, xylem pericycle development, pollen fertility, fruit enlargement and seed-setting (Brady 2019; Nafati et al. 2011; Wen et al. 2013; Yang et al. 2011; Zhang et al. 2020). The SMRs were identified later than ICK/KRPs and have limited studies over the past few years. Reports show that SMRs participate in blocking mitosis and inducing endoreplication in *Arabidopsis* trichome and sepal epidermis, negative regulating leaf size by restricting cell proliferation and increasing plants innate immunity partly through SA (Churchman et al. 2006; Hamdoun et al. 2015; Roeder et al. 2010).

In this study, a novel cucumber dwarf mutant with terminal flowers was identified and designated as *dw2*. Field phenotyping and cytological analysis showed that the dwarf phenotype was mainly resulted from significantly reduced internode number. We performed BSA-seq and map-based cloning to identify the gene controlling dwarf phenotype in *dw2*, and delimited the *dw2* locus to a 56.4 kb region containing five genes. Of all variations between W-pool and M-pool, the 7.9 kb deletion which resulted in *CsaV3_5G035790* complete deletion in *dw2* was more noticeable. Further haplotype analysis and gene expression analysis indicate that *CsaV3_5G035790* was the candidate gene responsible for the dwarf phenotype in *dw2*. RNA-seq analysis showed that a number of cyclins and several reported organ size regulators expressed differentially between WT and *dw2*, which may account for the reduced organ size in dwarf plants. In addition, the down-regulation of *CsSTM* and *CsWOX9* in *dw2* resulted in premature termination of SAM development, which eventually reduced the internode number and plant height.

## Materials and methods

### Plant materials

The dwarf mutant was first identified from the F_2_ population constructed with CG1601 (Northern China, monoecious) and CG3011 (Japan, monoecious) in 2018. Few self-pollination seeds were obtained due to the abnormal fruit development, while all of the offspring plants still displayed dwarf phenotype. One of these offspring plants was selected and designated as *Csdwarf2* (*dw2* hereafter), then *dw2* (pollen parent) was crossed with CG1601 (pistillate parent) to construct F_2_ population (Fig. S1). The F_2_ and the derived F_2:3_ populations were used for inheritance analysis and map-based cloning. Normal plants and dwarf plants from the derived F_2:3_ populations were used for phenotyping and gene expressing analysis.

### Mapping strategy and identification of the candidate gene of *dw2*

BSA-seq was used for mapping the candidate gene of *dw2*. The young leaves of 50 wild type (WT) and 50 dwarf individuals were sampled, respectively, for genomic DNA extracting with CTAB method (Murray and Thompson 1980). Pair-end sequencing libraries with a read length of 150 bp and insert sizes of 350 bp were subjected to whole genome re-sequencing with Illumina HiSeq 2500, and roughly 40 × genome sequences for each pool were generated. Short reads were aligned against the reference genome of cucumber inbred line “9930” using the Burrows–Wheeler Aligner (BWA) (Li and Durbin 2009), and alignment files were converted to SAM or BAM files using SAMtools (Li et al. 2009), and SNPs were identified using bcftools program. SNPs between the two pools were identified for further analysis when the base quality value was > 20 and the SNP quality value was > 20. The output of variant sites was introduced into a filter pipeline to minimize false positives caused by sequencing or alignment errors. The pipeline included several criteria: (1) A reliable SNP should be bi-allelic between W-pool and M-pool; (2) The base quality scores of both sequencing and read mapping should be higher than 20; (3) The number of uniquely mapped reads should be more than 3 and less than 120 at any SNP site.

Based on these criteria, we calculated a SNP index for both pools expressing the proportion of reads harboring SNPs that were identical to those in the M-pool; and obtained ΔSNP index by subtracting the SNP index for the W-pool from that for the M-pool. We calculated an average SNP index for the M-pool and W-pool using a 2500-kb sliding window with a step size of 20 kb; and then plotted the graph for the average of SNP-index and ΔSNP-index in W-pool and M-pool against the genome positions. Based on the re-sequencing data, dCAPS and CAPS markers were designed with dCAPS Finder 2.0 (http://helix.wustl.edu/dcaps/).

### Paraffin sectioning

Mature stems from wild-type and mutant plants were collected and fixed with FAA (50% ethyl alcohol, 5% glacial acetic acid, 2% formaldehyde) overnight at 4 °C. The materials were dehydrated with a graded series of ethanol (30–50–70–80–95–100%), infiltrated with xylene, and then embedded in paraffin (Sigma-Aldrich, USA). The 10-μm-thick sections were cut (Leica Microsystems, Wetzlar, Germany) and transferred onto poly-L-lysine-coated glass slides, deparaffinized in xylene and dehydrated through an ethanol series (100–95–80–70–50–30%), and stained with 0.1% toluidine blue. The samples were observed under a Leica DM5500B microscope.

### Scanning electron microscopy

The eighth leaves of WT and *dw2* were cut into pieces and fixed in 2.5% glutaraldehyde for 2 days and then, washed with PBS for three times. Afterward, the samples were dehydrated with gradient ethanol (50–70–80–90–95–100%), dried and coated with gold particles. The samples were examined with S-570 scanning electron microscope (HITACHI, Japan).

### Phylogenetic analysis

Multiple sequence (Gene ID shown in Table S4) alignment was performed using the muscle program with default parameters (Rodriguez-Leal et al. 2019). The sequence alignments were used for the subsequent phylogenetic analysis. The evolutionary history was inferred using the Neighbor-Joining method (Saitou and Nei 1987). The percentage of replicate trees in which the associated taxa clustered together in the bootstrap test (1000 replicates) are shown next to the branches (Felsenstein 1985). The evolutionary distances were computed using the p-distance method and are in the units of the number of amino acid differences per site. The analysis involved 20 amino acid sequences. All positions with less than 60% site coverage were eliminated. That is, fewer than 40% alignment gaps, missing data, and ambiguous bases were allowed at any position. There were a total of 112 positions in the final dataset. Evolutionary analyses were conducted in MEGA7 (Kumar et al. 2016).

### Subcellular localization

To explore the distribution of CsSMR1 in cells, the coding sequence of *CsSMR1* without the termination codon (primers are listed in Table S3) was cloned into pCAMBIA1300-GFP vector to generate 35S: CsSMR1-GFP vector. The recombinant expression vector was mixed with NLS-RFP vector and P19 and then injected into tobacco (*Nicotiana benthamiana*) leaves, with NLS-RFP vector and P19 injection as control. The injected tobacco plants were kept in darkness for 12 h and then cultivated under lighting conditions for 2 days and then, leaf samples were observed under laser scanning confocal microscope (LEICA TCS SP8).

### Chlorophyll content analysis

The total chlorophyll in the wild-type and *dw2* leaves was extracted with 95% ethanol and analyzed using a spectrophotometer (Shimadzu, Japan). The total chlorophyll, chlorophyll a, and chlorophyll b contents were estimated with light absorption values at 649 and 665 nm, respectively (Gregor and Maršálek 2004).

### Ploidy level analysis

0.2 g of WT and *dw2* leaves was placed in 500 μl nuclei extraction buffer (0.2 M Tris–HCl, 4 mM MgCl_2_, 2 mM EDTA, 86 mM NaCl, 10 mM Na_2_S_2_O_5_, 1% PVP-10, 1% Triton X-100, pH 7.5), chopped with sharp blade and extracted for 60 s, then filtered through a 50 μm filter. Followed by addition of 1600 μl of staining buffer for 60 s in dark. Nuclei suspensions were analyzed by CyFlow Space Flow Cytometer (Sysmex Partec, Muenster, Germany) and the corresponding FloMax software.

### RNA-seq analysis

The shoot apical of WT and *dw2* (30 days after planting) was used for RNA extraction and sequenced with Sanger/Illumina 1.9 platform. RNA-seq reads of six samples trimmed for quality and mapped onto the assembled genome “9930” using HISAT2 (Kim et al. 2015) with parameters “-*x*–dta”. StringTie (Pertea et al. 2015) were applied to compute expression level for each predicted gene in terms of FPKM (Fragments per Kilobase of Transcript per Million Mapped Reads) values using “-e -G” parameters. DESeq2 (Love et al. 2014) was used to normalize read counts and to test for differential expression. The differentially expressed genes (DEGs) between the bulks were identified with *P*-value < 0.05 and |log_2_(*f*c)|≥ 0.75 as the significance cut-off. Kyoto Encyclopedia of Genes and Genomes (KEGG) terms were determined with the online omicshare (https://www.omicshare.com/tools/Home/Soft/gogsea) website.

#### Determination of endogenous phytohormone content

IAA, ABA, CKT, GA, SA content detection. Young seedlings of WT and *dw2* were sampled and ground into a powder with liquid nitrogen, weigh and transfer each sample (1 g) to 15 mL screw-cap tubes, add 8 μL working solution (1 μg/mL) of internal standards to each 15 mL tube, then add 10 mL extraction solvent (2-propanol/H_2_O/concentrated HCl, 2:1:0.002, v/v/v) to each tube. Put the tubes on a shaker at a speed of 100 rpm for 30 min at 4 °C, add 5 mL dichloromethane to each sample and shake for 30 min at 4 °C, the centrifuge at 13,000* g* for 5 min. Transfer of the solvent from the lower phase using a Pasteur pipette into a screw-cap vial and concentrate the solvent mixture (not completely dry) using a nitrogen evaporator with nitrogen flow. The samples are redissolved in 0.4 mL methanol. Inject 2 µL of sample solution into the reverse-phase C18 Gemini HPLC column for HPLC–ESI–MS/MS analysis.

BR content detection. Young seedlings of WT and *dw2* were sampled and ground into a powder with liquid nitrogen, weigh and transfer each sample (1 g) to 15 mL screw-cap tubes, extracted in ice-cold 80% (v/v) methanol (10 mL) for 2 h. After centrifugation (4 °C, 10,000 rpm, 5 min), supernatant was extracted by Bond Elut Plexa SPE column (0.5 g, 6 mL, Varian, Palo Alto, CA, USA). The column was first conditioned with 10 mL 70% (v/v) ethanol and then equilibrated by 5 mL H_2_O and 5 mL 40 mM ammonium acetate (pH 6.5) and then, the sample was eluted by 3 mL methanol. Transfer of the solvent from the lower phase using a Pasteur pipette into a screw-cap vial and concentrate the solvent mixture (not completely dry) using a nitrogen evaporator with nitrogen flow. The samples are redissolved in 0.2 mL methanol. Inject 2 µL of sample solution into the reverse-phase C18 Gemini HPLC column for HPLC–ESI–MS/MS analysis.

## Results

### The phenotype and inheritance of *dw2* mutant

**Fig. 1:**
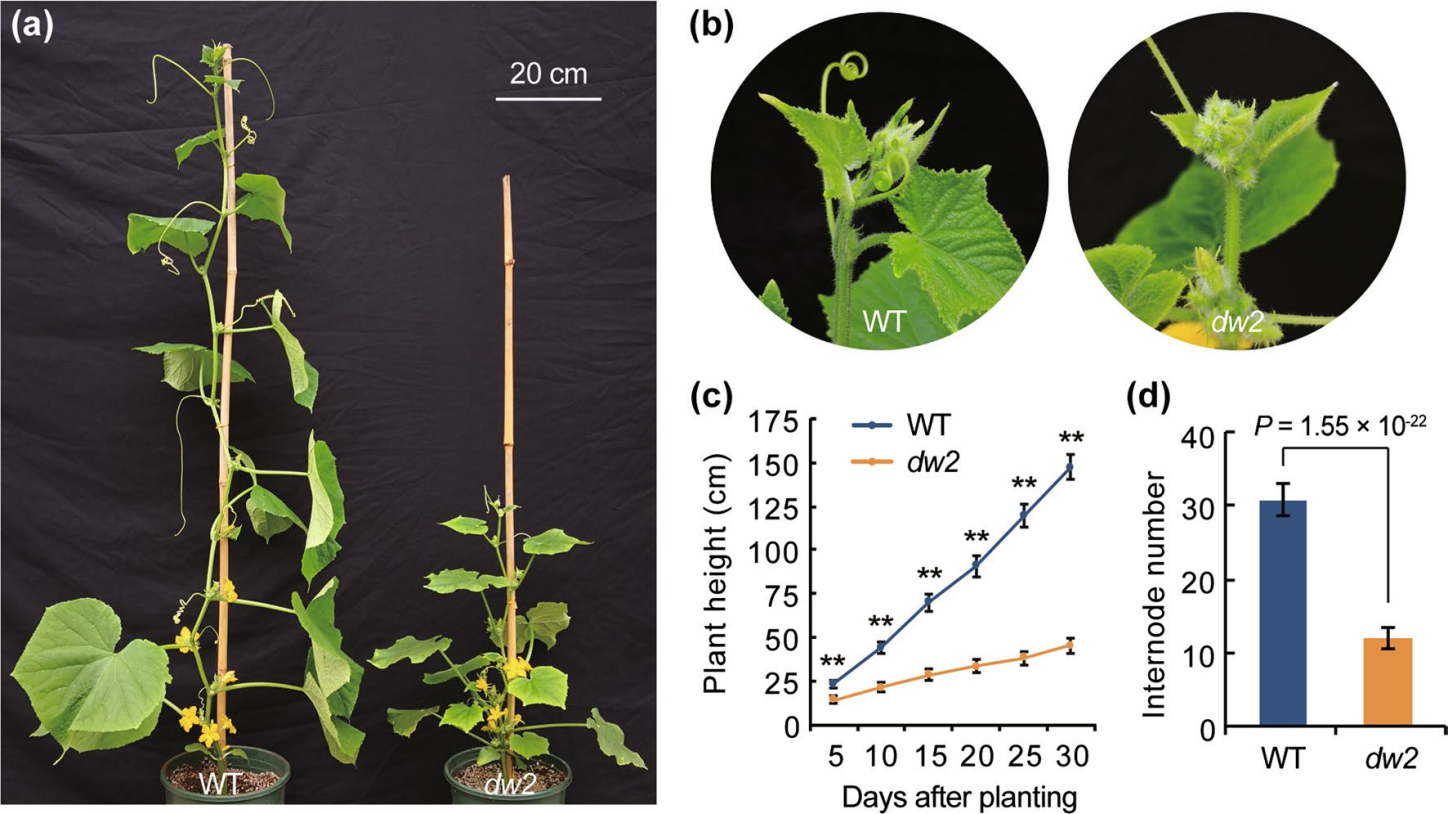
Phenotypic characterization of WT and the *dw2* plants. **a** Phenotype comparison between wild-type and *dw2* at adult period. Scale = 20 cm. **b** Shoot apical of WT and *dw2* at adult period. **c** Statistical data analysis of plant length between wild-type and *dw2* at different developmental stage. **d** Statistical data analysis of internodes number between wild-type and *dw2* at adult period. Error bar represents ± SD, *n* = 14, **P < 0.01 (Student’s *t*-test)

Compared with WT, the *dw2* mutant showed smaller and wrinkled leaf with yellow margin from the two true leaf stage, and plenty of axillary buds differentiated subsequently at each node (Fig. S2a, c). When plants grew up to adult stage (~ 30 days after planting), the height of *dw2* (45.1 ± 4.6 cm) was nearly one-third of plant height in WT (147.2 ± 7.0 cm) (Fig. 1a, c). Moreover, *dw2* harbored premature terminal flowers within its shoot apical, thus generated only 11.9 internodes in average, while 30.8 internodes for WT in average (Fig. 1b, d). Stem histological sectioning analysis revealed that *dw2* plants with reduced cell size than that of WT (Fig. S2b, e). Additionally, it was difficult to obtain self-pollination seeds of *dw2* plants as its fruit developed abnormally (Fig. S2d, f). These data indicate the dwarf phenotype in *dw2* is mainly due to terminal flowers which dramatically decreased plants internode number, and subsequently owing to the reduced cell size.

The cloning F_2_ population was developed by the cross between CG1601 (P1) as the pistillate parent and *dw2* as the pollen parent (P2). All F_1_ individuals displayed normal plant height as WT, indicating the dominance of normal plant height over the dwarf phenotype. Among the 300 plants in F_2_ population, 218 individuals exhibited normal plant height and 82 individuals displayed dwarf phenotype, which showed a segregation ratio of 3:1 (*χ*^2^ = 0.871, *P* > 0.05), implies that the dwarf phenotype in *dw2* was controlled by a single gene (Table S1).

### Fine mapping of the *Csdw2* gene

**Fig. 2:**
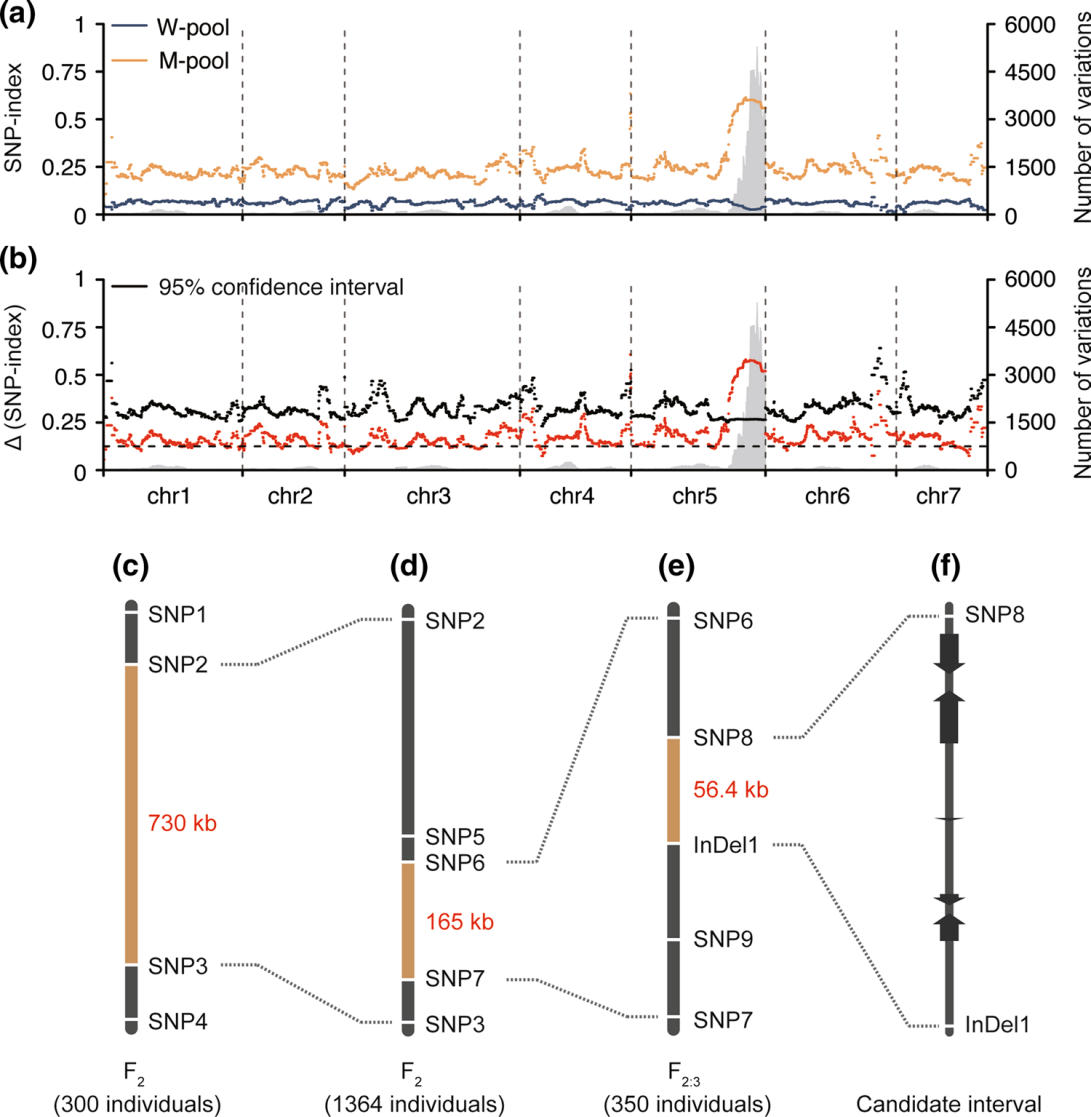
Map-based cloning of *dw2*. **a, b** BSA-seq analysis identified *dw2* locus in Chr5. **c** Primary mapping of *dw2* locus. **d, e** Fine-mapping of *dw2* locus. **f** Predicted genes in candidate interval

To identify the candidate region contributing to the dwarf phenotype in *dw2*, 50 normal and 50 dwarfism F_2_ plants were sampled, respectively, to construct W-pool and M-pool. We obtained 130 Gb and 140 Gb data for W-pool and M-pool and aligned W-pool (44 × depth; 99.13% coverage; 96.39% mapping rate) and M-pool (38 × depth; 99.11% coverage; 96.04% mapping rate) to the “9930” reference genome, respectively. Among these, approximate 484,035 SNPs between the two bulks were identified with the base quality value ≥ 20 and the SNP quality value ≥ 20. We plotted graph for W-pool and M-pool with average SNP-index and average ΔSNP-index using a 2500-kb sliding window with a step size of 20 kb. This resulted in a peak region between 21.36 to 31.91 Mb with a cluster of SNPs harboring high SNP-index (out of 95% confidence values) resided on chromosome 5, therefore, it was named *dw2* locus (Fig. 2a-b).

By applying molecular markers for genotyping in the F_2_ population, we primarily mapped *dw2* locus to a 730 kb interval between markers SNP2 and SNP3 (Fig. 2c). To narrow down the *dw2* region, an F_2_ population contains 1364 individuals that were genotyped with flanking markers SNP2 and SNP3, and 22 recombinants were identified. We subsequently genotyped these 22 recombinants with extra three markers (SNP5 ~ SNP7) between SNP2 and SNP3, the *dw2* locus was narrowed down to the 165 kb region between markers SNP6 and SNP7 (Fig. 2d). Afterwards, a F_2:3_ population containing 350 individuals derived from F_2_ recombinants was genotyped with another three markers (SNP8, SNP9 and InDel1). Finally, the *dw2* locus was narrowed down to a 56.4 kb region between markers SNP8 and InDel1 (Fig. 2e). There were five predicted genes in the 56.4 kb *dw2* locus, which were annotated as phosphoglucan phosphatase (*SEX4, CsaV3_5G035770*), UDP-glycosyltransferase (*UGT, CsaV3_5G035780*), cyclin-dependent protein kinase inhibitor SMR1-like (*SMR1, CsaV3_5G035790*), non-symbiotic hemoglobin 2 (*HB2, CsaV3_5G035800*) and RING/U-box superfamily protein (*CsaV3_5G035810*), respectively (Fig. 2f; Table 1).

**Table 1:** Annotation and variation of five genes in candidate region

Gene ID	Annotation	UTR	Intron	Exon	
				Synonymous	Non-synonymous
*CsaV3_5G035800*	Phosphoglucan phosphatase	0	0	0	0
*CsaV3_5G035800*	Glycosyl transferase	0	0	0	0
*CsaV3_5G035790*	CDK inhibitor SMR1-like	Completely deletion			
*CsaV3_5G035800*	Non-symbiotic hemoglobin 2	22	3	1	2
*CsaV3_5G035810*	RING/U-box superfamily	2	1	5	0

### Candidate genes analysis

**Fig. 3:**
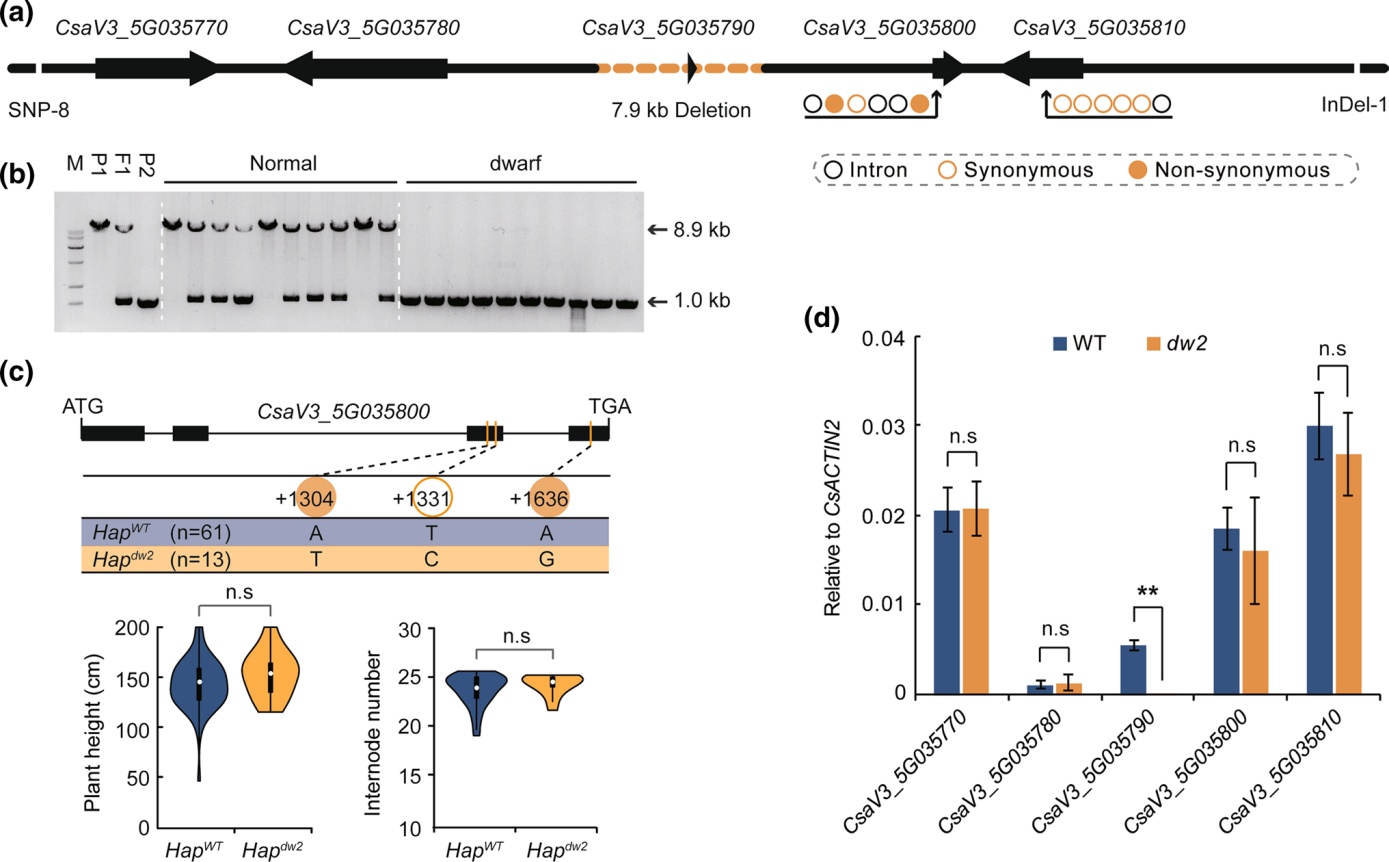
Candidate genes analysis. **a** Predicted genes and gene region variations in the 56.4 kb interval. **b** PCR validation of the 7.9 kb deletion. **c** Haplotype analysis and phenotype comparison of *CsaV3_5G035800*. **d** Expression levels of five predicted genes detected by qRT-PCR in WT and *dw2* plants. Error bar represents ± SD, *n* = 3, n.s indicates no significant difference, **P < 0.01 (Student’s *t*-test)

According to the whole-genome resequencing data of W-pool and M-pool, only three of these five genes have genomic sequence variation, including a 7.9 kb deletion and 35 SNPs (Fig. 3a; Table 1). Of these variations, the 7.9 kb deletion leads to the completely deletion of *CsaV3_5G035790* that was more noticeable (Fig. S3). We next designed specific primers to amplify the target fragment among 300 randomly selected F_2_ population individuals. There were 74 dominant homozygous plants, 142 heterozygous plants and 84 recessive homozygous plants which were exactly match the phenotype. Thus, the 7.9 kb deletion was proved to be co-segregated with the dwarf phenotype (Fig. 3b).

*CsaV3_5G035800* harbors 28 SNPs, only three SNPs located in coding regions, and one SNP resulted in a synonymous mutation (T1331C) and two SNPs resulted in non-synonymous mutations (A1304T and A1636G). To find out if the above-mentioned three coding region SNPs in *CsaV3_5G035800* caused dwarf phenotype in *dw2*, haplotype with 3 SNPs was conducted using 115 cucumber inbred lines (Qi et al. 2013). We identified two haplotypes (*Hap*^*WT*^ and *Hap*^*dw2*^) in 115 lines, and found no significant difference in plant height and internode number between *Hap*^*WT*^ and *Hap*^*dw2*^ (Fig. 3c; Table S5), indicating that *CsaV3_5G035800* was not responsible for the *dw2* phenotype. Similarly, we captured eight SNPs in *CsaV3_5G035810*, five SNPs resided in coding region; however, no one resulted in non-synonymous mutation (Table 1).

To further investigate the above annotated five genes may contribute to the *dw2* phenotype, we isolated RNA from shoot apical of WT and *dw2* to examine the transcription levels of these five genes and found only *CsaV3_5G035790* showed a striking difference between WT and *dw2* (Fig. 3d). Consequently, the consistency between the genomic sequence analysis and gene expression comparison reflected that *CsaV3_5G035790* is the candidate gene resulting in dwarf phenotype in *dw2*.

### Temporal and spatial expression patterns of the *CsSMR1*

**Fig. 4:**
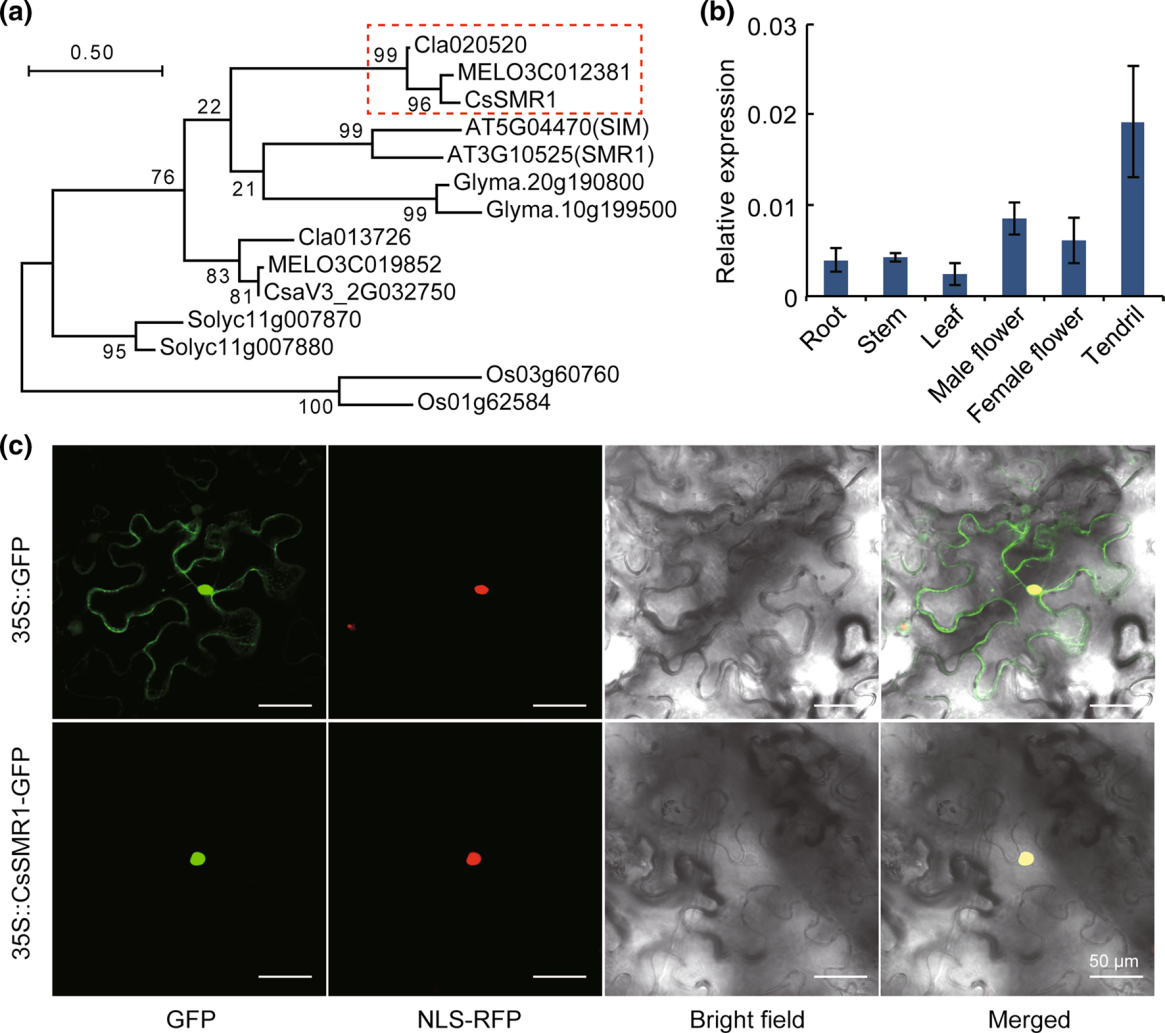
Temporal and spatial expression patterns of *CsSMR1*. **a** Phylogenetic analysis of CsSMR1 in cucumber and its homologs in other species. **b** Expression pattern analysis of *CsSMR1*. **c** The CsSMR1 localizes to the nucleus. Scale = 50 μm

According to the gene function annotation, *CsaV3_5G035790* encoded a SMR1-like Cyclin-dependent protein kinase inhibitor that functioned in mitosis and thus named as *CsSMR1*. Phylogenetic analysis revealed that CsSMR1 and its orthologs in melon and watermelon constitute a cucurbit-specific branch of the cyclin-dependent protein kinase inhibitor that belongs to the SIAMESE family (Fig. 4a). Transcriptomic data showed that *CsSMR1* was constitutively expressed in different tissues (roots, stems, young leaves, male flower, female flower and tendrils), indicating that *CsSMR1* may be involved in fundamental developmental process (Fig. 4b). Previous study found that SIM contains two NLSs at C-terminal that were required for its function (Kumar et al. 2018). Therefore, subcellular localization was conducted to explore the expression location of CsSMR1 in tobacco leaves. The fluorescence results of infected tobacco leaves displayed that the CsSMR1-GFP fusion protein was located in nucleus, while free-GFP was observed in the nucleus and cell membrane (Fig. 4c).

### The complete deletion of *CsSMR1* leads to decreased cell size in *dw2*

**Fig. 5:**
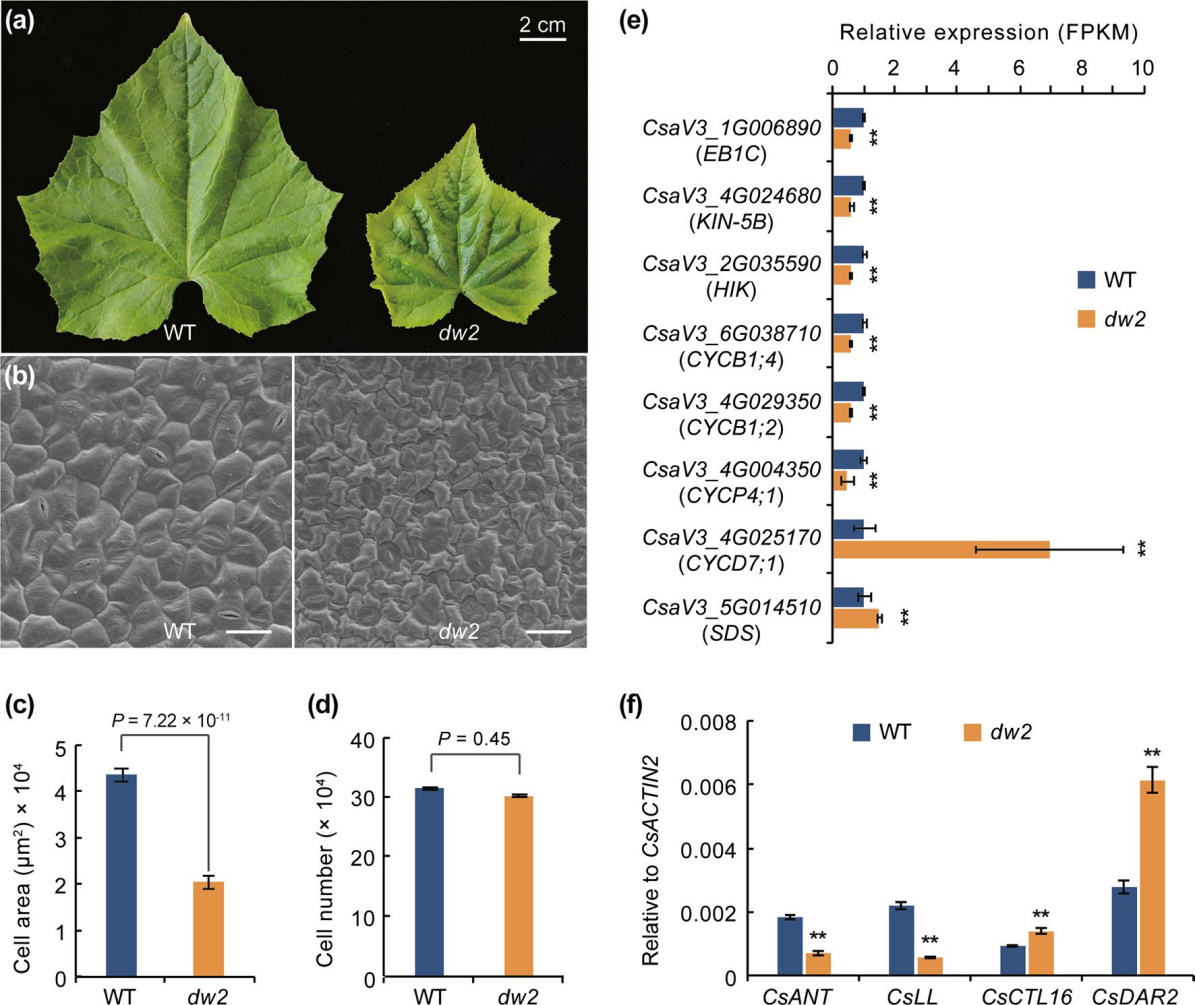
Cytological observation and organ size-related genes expression analysis of WT and *dw2*. **a** Leaf size was reduced in *dw2*. Scale = 2 cm. **b** SEM of eighth leaves in WT and *dw2*. Scale = 200 μm. **c, d** Calculated cell area and cell number in WT and *dw2*. **e** Expression level of expansion and cyclin genes in WT and *dw2*. **f** Expression comparison of previously reported genes which regulate organ size. Error bar represents ± SD, *n* = 3, **P < 0.01 (Student’s *t*-test)

As we mentioned above, the leaves and fruits size were dramatically reduced in *dw2*. To explore whether the reduction in organ size was due to decreased cell size or cell number, the adaxial side of 8th leaves was collected and fixed for scanning electron microscope. Cytological analysis showed that the *dw2* cells were about half size of WT, while the cell number showed no significant difference (Fig. 5a–d). The fruit of WT and *dw2* (12 days after pollination) were collected for paraffin sectioning, and statistical result shows that the *dw2* fruits cell size were evidently reduced than that of WT (Fig. S4a–c).

To explore the potential regulatory roles of *CsSMR1* referring to the formation of the dwarf in *dw2*, we performed RNA-seq analysis and obtained 1526 differentially expressed genes. Among these DEGs, we first screened several genes which are reported to regulate cell enlargement or cell proliferation, including five cyclins, two kinesin-like protein and a microtubule-associated protein, while most of these genes were down-regulated in *dw2* (Fig. 5e). Besides, four more genes which were reported to involve in regulating organ or cell size were found in DEGs. *AINTEGUMENTA* (*ANT*) encodes a putative transcriptional regulator and was previously demonstrated to participate in regulating organ size (Mizukami and Fischer 2000; Randall et al. 2015). *Littleleaf* (*LL*) encodes a WD40 repeat domain-containing protein and was revealed to integrate several known organ size regulators and associated pathways to control organ size (Yang et al. 2018). *BIG BROTHER* (*BB,* or *CTL19*) and *DA1*-related protein 2 (*DAR2*) are well-known negative regulator which control plant organ size during proliferative phase through ubiquitin–proteasome pathway (Disch et al. 2006; Peng et al. 2015). RNA-seq data showed that the expression of *CsANT* (*CsaV3_4G035300*) and *CsLL* (*CsaV3_6G009540*) was down-regulated in *dw2*, while *CsDAR2* (*CsaV3_5G008440*) and *BIG BROTHER* related gene *CsCTL16* (*CsaV3_6G034230*) was up-regulated in *dw2*, which were also confirmed with qRT-PCR (Fig. 5f). To examine whether plant hormones involved in the organ size regulating in *dw2*, young seedlings of WT and *dw2* were sampled to conduct phytohormone content measurement. Result showed that most of the detected plant hormones content were decreased in dwarf plants, including IAA, TRZ (a kind of CTK), GA_4_, ABA, 6-DCS (precursor of BR) and SA (Fig. S6a). Besides, KEGG analysis showed that the biosynthesis and metabolism of vital primary and secondary metabolites were significantly enriched (Fig S6b). In summary, the involvement of several reported regulators and reduced of plant hormone content may result in the dwarf phenotype in *dw2*.

### Down-regulation of *CsSTM* and *CsWOX9* results in premature SAM termination in *dw2*

**Fig. 6:**
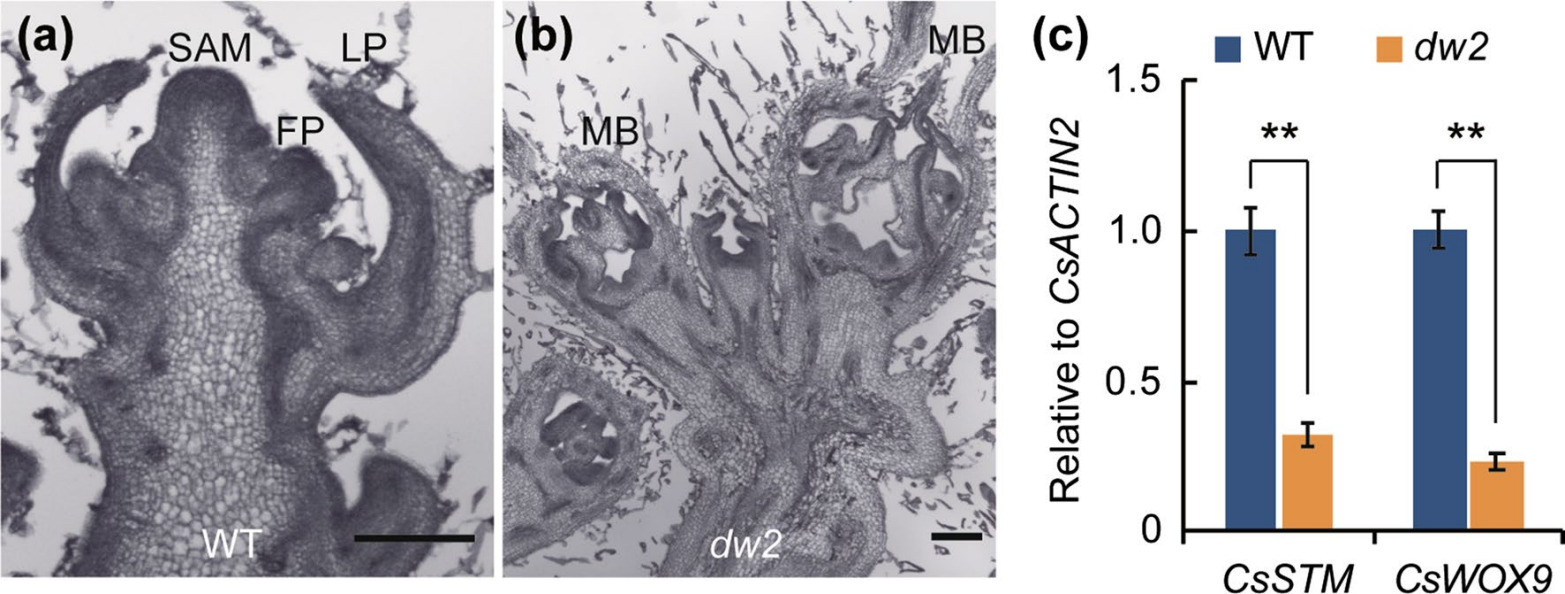
Down-regulated of *CsSTM* and *CsWOX9* results in SAM termination in *dw2*. **a, b** Histology observation of the changes at shoot apexes of WT and *dw2* plants at 25 days after planting. LP, leaf primordia; FP, floral primordia; MB, male bud. Scale = 200 μm. **c** *CsSTM* and *CsWOX9* was down-regulated in *dw2*. Error bar represents ± SD, *n* = 3, **P < 0.01 (Student’s *t*-test)

To test if *CsSMR1* had any effect on the SAM development, paraffin section was conducted with shoot apical of WT and *dw2* at 25 days after planting. Consistent with phenotype, the WT plants develop with normal shoot apical meristem, while *dw2* plants exhibited premature termination of shoot apical meristem (Fig. 6a, b). As the marker genes of SAM, *SHOOTLESS* (*STM*), *WUSCHEL* (*WUS*) and several *WUSCHEL-LIKE HOMEOBOX*s (*WOX*s) are indispensable for the maintaining of SAM activity (Endrizzi et al. 1996; Hendelman et al. 2021; Laux et al. 1996). Accordingly, shoot apical of WT and *dw2* was sampled to examine the expression of *CsSTM*, *CsWUS* and *CsWOX9*. As expected, *CsSTM* and *CsWOX9* were significantly down-regulated in *dw2* (Fig. 6c). Accordingly, the complete deletion of *CsSMR1* in *dw2* decreased the expression level of *CsSTM* and *CsWOX9*, and then resulted in SAM size shrinking until termination.

## Discussion

### Identification of a novel dwarf mutant with determinate growth habit in cucumber

Cucumber is one of the most popular vegetables all over the world, suitable plant architecture varieties are often selected for different production systems. Varieties with indeterminate growth habit are often grown under protected environments, for the fruits can be harvested continuously for an extended growth period; however, varieties with determinate growth habit and compact plant architecture are often selected under open fields, for it can be harvested once-over and planting with high-density. To date, seven dwarf mutants have been characterized and map-based cloned with forward genetics approach. Among these mutants, *compact* (*cp*), *cucumber dwarf* (*Csdw*), *super scp-1* (*scp-1*), *super scp-2* (*scp-2*) and *compact plant architecture* (*cpa*) were reported to play important role in the biosynthesis of cytokinin, gibberellin and brassinosteroid, respectively (Hou et al. 2017; Li et al. 2011; Wang et al. 2017; Xu et al. 2018; Zhang et al. 2021); *short internode* (*si*) participates in a wide range of physiological processes, and could influence multiple traits including internode elongation (Lin et al. 2016); *determinate* (*de*) and *determinate-novel* (*det-novel*) encode CsTFL1 and CsCEN respectively, and were confirmed to control cucumber determinate growth habit (Njogu et al. 2020; Wen et al. 2019, 2021).

In the current study, we identified a novel dwarf mutant with terminal flowers. We show that the strikingly reduced plant height in *dw2* was mainly resulted from premature termination of SAM and following dramatically decreased internode number (Figs. 1a–d, 6a, b). In addition, the dwarf plants exhibit reduced leaf area and abnormal fruit development (Fig. S2c–f). Using BSA-seq method combined with map-based cloning strategy, the *dw2* locus was delimited to a 56.4 kb region which contain five predicted genes (Fig. 2). Subsequently genomic variations analysis combined with genes expression analysis confirmed that the 7.9 kb deletion to be the causative variation responsible for the dwarf phenotype in *dw2* (Fig. 3). Accordingly, *CsaV3_5G035790*, the only gene in the 7.9 kb was considered as the candidate gene controlling *dw2* dwarf phenotype. It is different from all the dwarf mutants that have been reported previously.

### Cell size rather than cell number reduction is responsible for decreased organ size in *dw2*

Coordination between cell division and growth is required for proper development of multicellular organisms. CDK inhibitors (CKIs) negatively regulate cell cycle progression by binding to and inhibiting D-type CYC and A-type CDK subunits (Inzé 2005). There were two types of plant-specific CKI in land plants, known as Kip-related proteins (KRP) and SIAMESE (SIM), respectively. In our study, we demonstrate that *Csdw2* encodes an ortholog of *Arabidopsis* SIM/SMR1 and is the key regulator of organ size in cucumber. In the *CsSMR1*^*del*^ plants, reduced cell size rather than cell number resulted in decreased organ size (Fig. 5a–d). Several cyclins and kinesin-like proteins were found to expressed differentially between WT and *dw2*, implied that they may involve in the regulation of cell differentiation (Fig. 5e). In addition, several reported genes that involved in regulating organ size were expressed differentially between WT and *dw2*. *CsANT* and *CsLL*, two positive regulators controlling organ size, were down-regulated in *dw2*; two negative regulators in the ubiquitin–proteasome pathway, *DAR2* and *BB*-related genes (*CTL16*), were up-regulated in *dw2* (Fig. 5f).

In *Arabidopsis*, SIM and SIM-related genes regulate the cell size and organ size by inducing endoreplication, which blocking cells entry into mitosis and hence increased the DNA content. Overexpression and knocking out of *SIM* or *SMR*s increased and reduced the ploidy level respectively (Churchman et al. 2006; Roeder et al. 2010). However, we found the ploidy level between WT and *CsSMR1*^*del*^ plants showed no significant difference (Fig. S5a-c). SMRs is a relatively large family in plants, as there are 17 members in *Arabidopsis* and over 12 members in cucumber (Kumar et al. 2015). It was reported that most of SMRs share similar fundamental functions during mitotic (Kumar et al. 2018; Yi et al. 2014). Among the DEGs between WT and *dw2*, we found *CsaV3_5G031210* (*CsSMR2-like*) was upregulated about three-fold over WT in *CsSMR1*^*del*^ plants and it was confirmed with qRT-PCR (Fig. S5d). We speculate that the transcriptional compensation mechanism led to the difference between cucumber and *Arabidopsis*. In fact, the transcriptional compensation mechanism is very common among animals and plants. For instance, knocking out of *Capn3a* in zebrafish did not exhibit a small liver phenotype, which was proved to in consequence of the significantly upregulated of other 10 members (Ma et al. 2019). *SlCLE9* was upregulated for more than 40-fold in *slclv3* meristems, which accounting for the increasing of meristem size (Rodriguez-Leal et al. 2019). Consequently, we believe that the upregulation of *CsSMR2-like* to a certain extent compensate for the complete loss of *CsSMR1* in *dw2*.

### Possible mechanisms of *CsSMR1*-mediated SAM development control in cucumber

Plant hormones are independent and coordinate with each other in regulating almost every aspect of plant growth and development process. Among them, cytokinin is the key hormone that involved in regulating SAM development. The SAM size was reported to correlate with the expression level of *CYCD3*, which is reduced by exogenous cytokinin treatment (Riou-Khamlichi et al. 1999). WUS can directly repress the cytokinin signaling negative regulators (*ARRs*) to maintain meristem activity (Hwang and Sheen 2001). The overexpression of *catabolic enzyme cytokinin oxidase 1* (*CKX1*) in tobacco plants decreases the endogenous cytokinin content, producing stunted shoots with smaller SAM (Werner et al. 2001). As we mentioned above, most of the detected plant hormones content were decreased in dwarf plants (Fig. S6a). Additionally, KEGG pathway analysis was conducted with DEGs between WT and *dw2*, zeatin biosynthesis (KO00908) was significantly enriched (Fig. S6b). Exogenous auxin treatment could induce the expression of *WUS*, and this induction is essential for the embryonic stem cell self-renewal during somatic embryogenesis (Su et al. 2009). Moreover, plant hormones may function synergistically or antagonistically in regulating the SAM activity. *STM* could repress GA biosynthesis through activate CK biosynthesis and signaling pathway, thus promoting meristem activity (Jasinski et al. 2005). According to KEGG analysis, plant hormone signal transduction (KO04075) was also enriched. Plenty of genes participate in biosynthesis, metabolism and signaling of multi-kind of plant hormones (Fig. S6c). These results indicate that multi-kind of phytohormones were integrated in regulating dwarf architecture in *dw2*.

Studies in *Arabidopsis* have revealed a CLV-WUS feedback loop that plays a central role in maintaining SAM activity. Our results showed that the down-regulation of *CsSTM* and *CsWOX9* resulted in early termination of SAM in *dw2*, hereby decreased internode number and plant height (Fig. 6). Nevertheless, how *CsSMR1* affects the expression of SAM-maintaining genes remains unclear. The *SELF-PRUNING* (*SP*) is the tomato functional homolog of *CEN* and *TFL1* in *Antirrhinum* and *Arabidopsis* respectively, which maintain the indeterminate state of inflorescence meristems (Pnueli et al. 1998). SELF-PRUNING INTERACTING PROTEIN4 (SIP4) was isolated in a Y2H screen using the tomato SELF-PRUNING (SP) protein as bait, which subsequently found to be the homolog of *SIM* (*Solyc11g007880*) and was shown to specially express in apical meristem of young seedling (Pnueli et al. 2001). *Solyc11g007880* shares an extent sequence similarity with CsSMR1 and also clustered with SIM/SMR1 branch (Fig. 4a). Given that one of the first events classically observed in the transition from vegetative growth to reproductive growth is a mitosis increase in the meristem (Steeves and Sussex 1989), it is reasonable to presume that *SIM* or *SIM*-related genes could be involved in regulating mitotic cycling during this developmental transition. However, the involving mechanism need to be further elucidated in the following study.

## Supplementary Information

Below is the link to the electronic supplementary material.

Supplementary file1 (PDF 10815 KB)

## Data Availability

The materials presented in this article will be freely available to any researcher wishing to use them for non-commercial purposes, and the author responsible for distribution of materials in accordance with the policy described in the Instructions for Authors (www.springer.com) is: Zhonghua Zhang (zhangzhonghua@caas.cn).
