# Supplementary material for: Deletion of a cyclin-dependent protein kinase inhibitor, CsSMR1, leads to dwarf and determinate growth in cucumber (Cucumis sativus L.)
Source: Theor Appl Genet. 2021 Nov 29;135(3):915–27. doi: 10.1007/s00122-021-04006-7 (PMC8942921; doi:10.1007/s00122-021-04006-7)
Supplement: Supplementary file 1 — Supplementary file1 (PDF 10815 KB) [file 122_2021_4006_MOESM1_ESM.pdf]

Supplementary Information

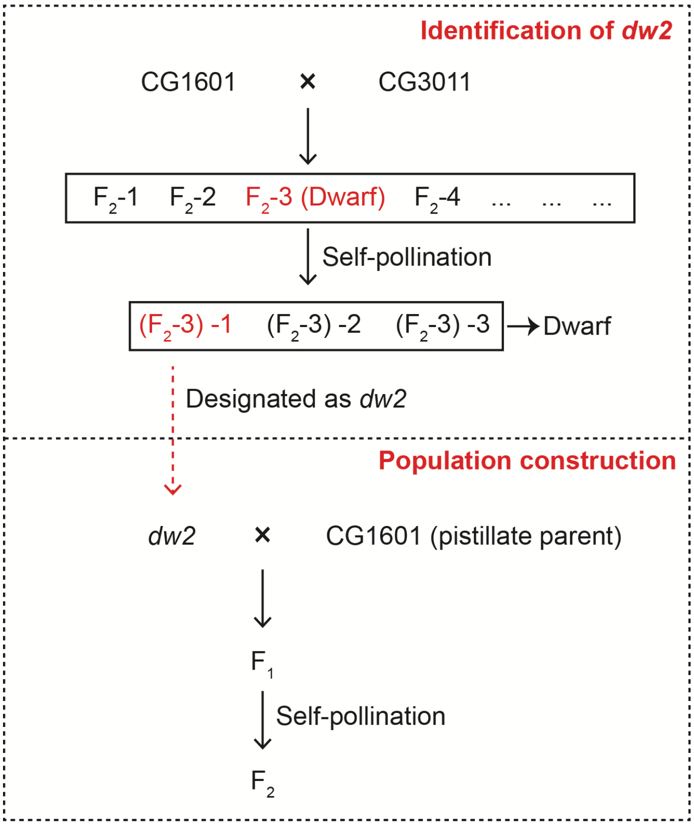

Supplementary Fig. S1 Identification of *dw2* mutant and mapping-population construction

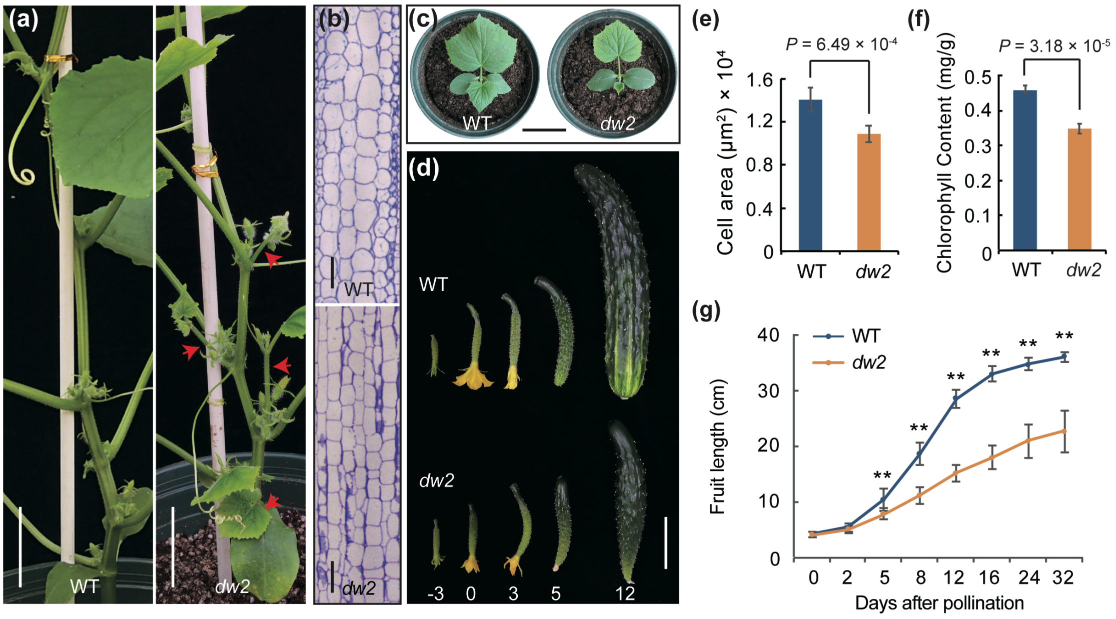

Supplementary Fig. S2 Phenotype characterization of WT and *dw2*

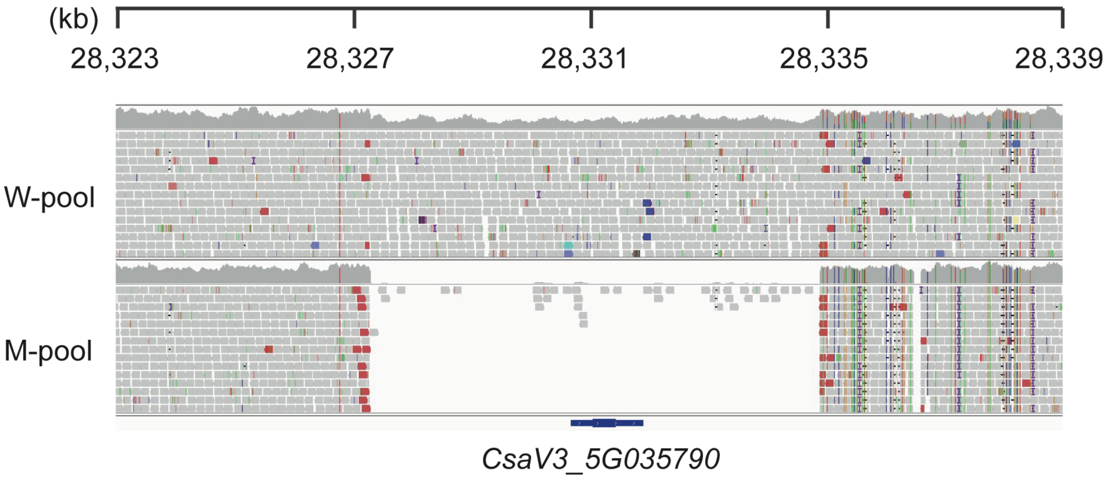

Supplementary Fig. S3 IGV visualization of 7.9 kb deletion in *dw2*

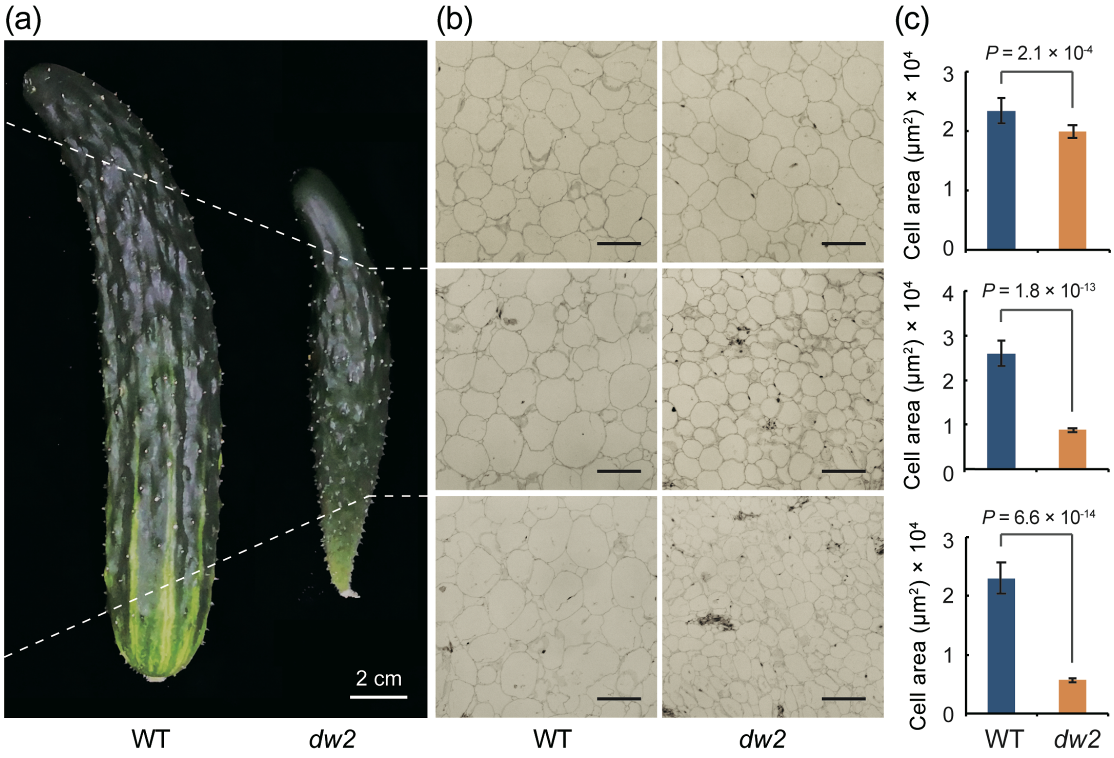

Supplementary Fig. S4 Cytological analysis of WT and *dw2* fruits

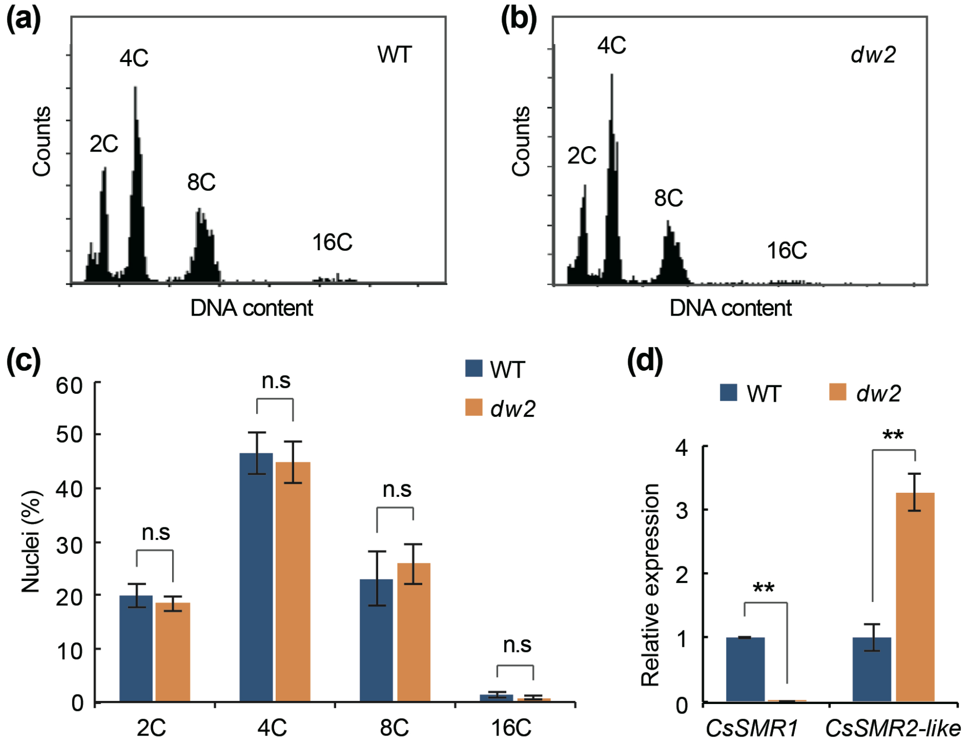

Supplementary Fig. S5 Ploidy level comparison between WT and *dw2*

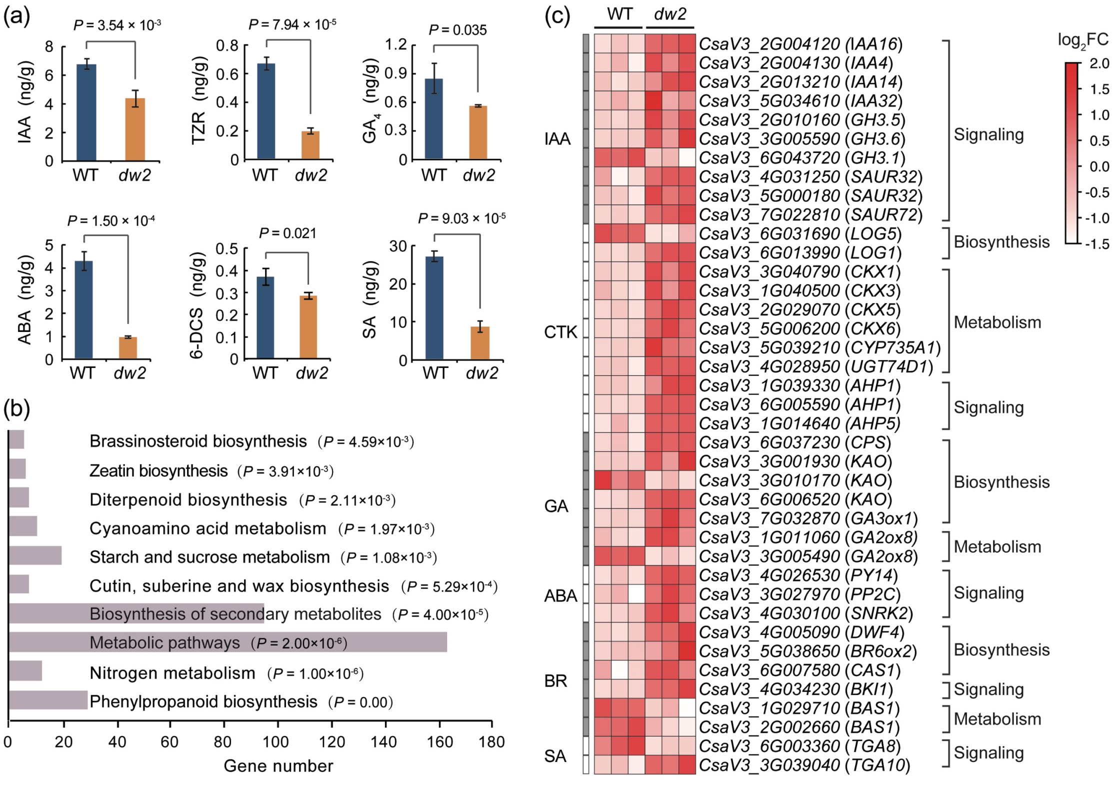

Supplementary Fig. S6 Biosynthesis of multi kind of plant hormone were affected in *dw2*

Supplementary Table S1. Inheritance analysis of *dw2* mutant

Generation	Population	Normal	Dwarf	Expected Ratio	χ^2	*P*-value
F_1	30	30	0	-	-	-
F_2	300	218	82	3:1	0.871	> 0.05

Supplementary Table S2 Primers for map-based cloning

Annotation	ID	Type	Sequence (5' to 3')
Mapping	SNP1	dCAPS (*Eco*R I)	AAACAGCTTCTGCAGCCGCGCAGCGGTGGA
			GTCTCTTCTTAGAGAGAAAATGG
	SNP2	dCAPS (*Mae* I)	GAAAAGTATTGAAATTGGGCAGTCAACCTA
			CTATGATATATTTACACTTTGC
	SNP3	dCAPS (*Nhe* I)	TAACAAGATGGGGAAGCTCATTTC
			AGAGAGTGAGAGCTTAGTCGTTGCGCTAG
	SNP4	CAPS (*Eco*R I)	AGGGAATTACGATTACAATGAAGTG
			CAGAACAAAGAATATTTCCGTAC
	SNP5	CAPS (*Mnl* I)	AAATTTAGGGAGAGAGATAGTAAAGATAC
			TTTTTGCAGTATGGACTTCCCAC
	SNP6	dCAPS (*Hin*d III)	GTAAAATCCAAATTAGTCAAACTATATAAG
			CGTAGAAGTACGTTGCAATCA
	SNP7	dCAPS (*Kpn* I)	AAGCTTGAAATGATGGGAATGTCCT
			TGTTCTCCACTGACAAAACAACTCGGTAC
	SNP8	dCAPS (*Eco*R I)	TTTAATAATTAGAAATACATTTTGGGAATT
			TCAATAAATGAGTAAAAACACAACG
	SNP9	dCAPS (*Eco*R I)	GAGCTCATTGGGTTCCAAAAGTAGT
			CTGGTGCTAATGCTGCTGATGAAGTGAAT
	InDel1	InDel	ATGCTCTGTGTGGCTTTGTATTTT
			GTTTAATGAACGAGGCTGGTGTAA
	SV	InDel	GTATAAGTGTCCACGATGTTTGTCC
			ACATTAGGGGTGGAGAATTTGAAGA

Supplementary Table S3 Primers for qRT-PCR and subcellular localization

Primer	Primer ID	Sequence (5' to 3')
qRT-PCR	CsaV3_5G035770	AAACGCTCATGCTTCCCAAAAATA
		CCCATCCAATATCAAGTCCAGCTA
	CsaV3_5G035780	TTGCTCTGAACATGAAGGAACTTG
		GGCTTAAAAGAACAAGTCGACCAA
	CsSMR1	GAAGAATTGAGTTGCCGTACACC
		TCTCTATGCACGATCTCCACAAAT
	CsaV3_5G035800	ATCCCTCAGAACAATCCTAAGCTC
		ATGACTCCATTACGAAGGTGAACA
	CsaV3_5G035810	TGATGCTGTAAATAGACCCACTCT
		AATCTCCCATTCAATTTCCGAACG
	CsWOX9	ATTAATATGTCCACTATTTCCACGCC
		AAACCCACACCATTGATGAATACG
	CsSTM	CGGGGTTAGATCAAAAGCAAATCA
		CCAAGGGAAAAGGATTCCCTAAGA
	CsSMR2-like	ATTACGACCTCCTCTATTGCTCAC
		ACGAAGATTTAGGCTTCTTAGGGG
	CsANT	AAAACGCGGAGCGATATTATTCTG
		CCGCTGCTCCATATCTTGTCTTAT
	CsLL	ATGAGACTTACATAGTGGTGGACG
		AGAACTCATCACAGCATAACCCAT
	CsCTL16	GGAGACTACGGATGGAAATGATGA
		CCAATGAACTCCCCTAATGCAATC
	CsDAR2	GTTCTTTATGGTCTCCCAAGATTACT
		TGATTCAAGCCACATGTAGGAAAG
Subcellular localization	CsSMR1	TCGACTCTAGTCTAGAATGTCCACAGAACTCGATCTCC
		CCCTTGCTCACCATGGTACCTTTACACTCGCAGAAGCTTCGTTTA

Supplementary Table S4 Gene ID for phylogenetic tree analysis

Species	Gene ID
Cucumis sativus	CsaV3_5G035790
	CsaV3_2G032750
Cucumis melo	MELO3C012381
	MELO3C019852
Cucumis lanatus	Cla020520
	Cla013726
Arabidopsis thaliana	AT3G10525
	AT5G04470
Glycine max	Glyma.20g190800
	Glyma.10g199500
Solanum lycopersicum	Solyc11g007870
	Solyc11g007880
Oryza sativa	Os03g60760
	Os01g62584

Supplementary Table S5 Germplasm resources used for haplotype analysis

**Germplasm**	**Haplotype**	**Plant height (cm)**	**Node number**
9930	Hap^{WT}	147	24
CG0001	Hap^{WT}	46	19
CG0002	Hap^{WT}	NA	NA
CG1043	Hap^{WT}	115	22
CG1071	Hap^{WT}	123	23.8
CG1077	Hap^{WT}	145	23
CG1083	Hap^{WT}	148	19.4
CG1247	Hap^{WT}	132.5	24
CG1292	Hap^{WT}	146.25	24.75
CG1322	Hap^{WT}	136	24
CG1373	Hap^{WT}	153	23.8

CG1541	*Hap*^{WT}	169	24.8
CG1601	*Hap*^{WT}	130	21
CG1602	*Hap*^{WT}	170	22.4
CG1697	*Hap*^{WT}	95	20.5
CG1778	*Hap*^{WT}	133	25
CG1811	*Hap*^{WT}	140	23.2
CG1876	*Hap*^{WT}	161	24
CG3007	*Hap*^{WT}	199	25.6
CG3010	*Hap*^{WT}	150	25
CG3127	*Hap*^{WT}	161.67	19
CG4041	*Hap*^{WT}	130	22.67
CG4210	*Hap*^{WT}	166	25
CG4353	*Hap*^{WT}	200	25
CG4354	*Hap*^{WT}	192	25
CG4357	*Hap*^{WT}	170	22.4
CG4360	*Hap*^{WT}	148	24.8
CG5031	*Hap*^{WT}	140	25
CG5071	*Hap*^{WT}	145	25
CG5234	*Hap*^{WT}	136.25	24
CG5326	*Hap*^{WT}	125	23
CG5420	*Hap*^{WT}	146	25
CG5756	*Hap*^{WT}	127	25
CG5790	*Hap*^{WT}	133	23.8
CG5801	*Hap*^{WT}	175	21
CG6508	*Hap*^{WT}	130	25
CG6562	*Hap*^{WT}	127	24.8
CG6578	*Hap*^{WT}	115	25
CG6586	*Hap*^{WT}	124	22.6
CG6600	*Hap*^{WT}	163	23.4
CG6601	*Hap*^{WT}	159	23.2
CG6647	*Hap*^{WT}	147	25
CG6663	*Hap*^{WT}	125	25

CG7086	*Hap*^{WT}	120	23.4
CG7704	*Hap*^{WT}	153.75	25
CG8039	*Hap*^{WT}	168.75	25
CG8093	*Hap*^{WT}	160	24.2
CG8099	*Hap*^{WT}	154	25
CG8191	*Hap*^{WT}	158	25
CG8198	*Hap*^{WT}	155	21.8
CG8216	*Hap*^{WT}	135	25
CG8916	*Hap*^{WT}	158	24.6
CG9143	*Hap*^{WT}	128	24.2
CG9153	*Hap*^{WT}	132	20.8
CG9160	*Hap*^{WT}	NA	NA
CG9165	*Hap*^{WT}	122	23
CG9185	*Hap*^{WT}	120	24.67
CG9187	*Hap*^{WT}	162	24.8
CG9201	*Hap*^{WT}	122.5	22.5
CG9203	*Hap*^{WT}	105	22.8
CG9207	*Hap*^{WT}	NA	NA
CG3076	*Hap*^{dw2}	200	25.2
CG3079	*Hap*^{dw2}	164	25
CG4359	*Hap*^{dw2}	177.5	25
CG5278	*Hap*^{dw2}	115	25
CG5670	*Hap*^{dw2}	170	22.67
CG7023	*Hap*^{dw2}	164	24.6
CG7744	*Hap*^{dw2}	125	25
CG7747	*Hap*^{dw2}	155	24.5
CG7748	*Hap*^{dw2}	131.25	21.6
CG8163	*Hap*^{dw2}	153.33	24
CG8712	*Hap*^{dw2}	158	25
CG8724	*Hap*^{dw2}	142.5	24
CG9164	*Hap*^{dw2}	135	23.5
